# Development of a Global Physical Literacy (GloPL) Action Framework: Study protocol for a consensus process

**DOI:** 10.1371/journal.pone.0307000

**Published:** 2024-08-12

**Authors:** Johannes Carl, Emiliano Mazzoli, Alexandre Mouton, Raymond Kim-Wai Sum, Amika Singh, Marlen Niederberger, João Martins, Dean Kriellaars, Nigel Green, Peter Elsborg, Dean A. Dudley, John Cairney, Jaime Barratt, Lisa M. Barnett

**Affiliations:** 1 Institute for Physical Activity and Nutrition (IPAN), School of Health and Social Development, Deakin University, Geelong, Victoria, Australia; 2 Department of Physical Activity and Rehabilitation Sciences, University of Liège, Liège, Belgium; 3 Department of Sports Science and Physical Education, Chinese University of Hong Kong, Shatin, Hong Kong; 4 Mulier Instituut, Utrecht, The Netherlands; 5 Department of Movement, School & Sports, Windesheim University of Applied Sciences, Zwolle, The Netherlands; 6 Department of Sports Science and Physical Education, University of Education, Schwäbisch Gmünd, Germany; 7 Centro de Estudos em Educação, Faculdade de Motricidade Humana e UIDEF, Universidade de Lisboa, Cruz Quebrada, Portugal; 8 Department of Physical Therapy, University of Manitoba, Winnipeg, Canada; 9 International Physical Literacy Association, Wigan, England, United Kingdom; 10 Center for Clinical Research and Prevention, Copenhagen University Hospital – Bispebjerg and Frederiksberg, Frederiksberg, Denmark; 11 Macquarie School of Education, Macquarie University, North Ryde, New South Wales, Australia; 12 School of Human Movement and Nutrition Sciences, University of Queensland, St. Lucia, Queensland, Australia; 13 Department of Educational Studies, Brock University, St. Catharines, Ontario, Canada; University of Malta, MALTA

## Abstract

**Background:**

The holistic concept of physical literacy (PL) has gained growing attention in recent research, policy, and practice. Many important policy documents of the physical activity and educational fields (e.g., Global Action Plan on Physical Activity 2018–2030 by the World Health Organization, UNESCO’s Quality Physical Education guidelines for policymakers) have specified PL. However, a clear framework for action is needed, as most initiatives across the world are fragmented, lack a prospective orientation, can benefit from conceptual clarification, and are not linked to effective translation into practice. Therefore, we aim to consensually develop a Global Physical Literacy (GloPL) Action Framework to define goals and principles (asking *what* is needed) as well as actions and ways (asking *how* these can be achieved) to move PL forward.

**Materials and methods:**

We apply a three-stage group Delphi technique involving three representation groups: (a) geographical representatives to achieve global coverage of perspectives; (b) representatives of special thematic interest reflecting prominent gaps of current PL activities; and (c) representatives of societies from the broad field of physical activity and health to facilitate dissemination. The process will begin with an individual pre-Delphi exercise, in which experts generate initial ideas for the framework, followed by a four-eye document analysis to derive themes for the discussion. Subsequently, the experts will meet face-to-face in three online rounds to discuss and prioritize the themes. Interspersed formal voting with pre-defined agreement thresholds (via descriptive statistics) will inform the inclusion of themes within the final framework.

**Conclusions:**

A global consensus on goals, principles, actions, and ways for the development of PL has the potential to provide a largely accepted roadmap for future activities in research, policy, and practice. The co-production approach will help disseminate the GloPL Action Framework and benefit work in relevant application fields of physical activity and health worldwide.

## 1. Introduction

### 1.1 The physical literacy concept

In recent years, researchers, practitioners, and policymakers have increasingly discussed the concept of physical literacy (PL) [1]. The PL concept adopts a holistic approach toward physically active lifestyles by integrating different individual determinants for physical activity [2–4]. These determinants span physical, affective, cognitive, and sometimes social or spiritual factors [2, 5–8], depending on the corresponding definition selected [9, 10]. Among the various conceptualizations published, the International Physical Literacy Association (IPLA) and the consensus-based Canadian approach, for instance, define PL as *“the motivation*, *confidence*, *physical competence*, *knowledge and understanding to value and take responsibility for engagement in physical activities for life”* [6, 11]. The Australian Physical Literacy Framework (APLF) defines PL as the *“Physical literacy is lifelong holistic learning acquired and applied in movement and physical activity contexts*. *It reflects ongoing changes integrating physical*, *psychological*, *social and cognitive capabilities”* (p. 5) [12]. More recently, China has specified PL as the “integration of physical, perceptual, cognitive, psychological, and behavioral capabilities, echoing with the need for an active, healthy, and fulfilling lifestyle, which involves continuous positive interactions with the environment and embodied engagement in physical activities for life” (p. 245) [13]. Finally, Sport England [14] has released its own understanding of the concept, with PL expressing individuals “own relationship with movement and physical activity throughout life” [14]. Irrespective of the slight nuances between some definitions culminating in a differential prioritization of domains (e.g., “social” in Australia or “perceptual” in China), the original PL conceptualizations grounded on recognized philosophical assumptions about human life, requesting analyses of physical activity to consider the inseparability of body and mind (monism/embodiment), the connectedness to the environment (existentialism), as well as the authenticity of individual perceptions and biographies (phenomenology) [15–17]. Importantly, these assumptions hold pivotal implications by questioning one-sided intervention approaches and prioritizations of single determinants (e.g., psychomotor aspects) [18]. PL largely detaches from external performance standards and instead favours a person-centred view on physical activity [15, 19]. Cognizant of the postulated value of PL for biopsychosocial health [3, 20], the concept holds an inviting and inclusive gesture toward each individual by emphasizing individual, lifelong growth—independent from constitution, age, and capabilities [21–24]. Accordingly, services in the spirit of PL require specific qualifications and skills among their deliverers, such as therapists, sport coaches, or physical education teachers [25–28].

Given the immanent asset to transport a holistic and inclusive message for physical activity, PL has the potential to permeate or enrich numerous application fields in the broad physical activity and health sphere, including, among others, physical education, sport, public health, health care/promotion, rehabilitation, recreation, an community services [10, 29, 30]. In fact, PL has entered many crucial international documents of the field. For instance, PL has at multiple positions been included into the Global Action Plan on Physical Activity 2018–2030 (GAPPA) by the World Health Organization [31], advocating its role to “raise awareness and knowledge of the health benefits of physical activity, promote behaviour change and increase health and physical literacy” (p. 63). Moreover, the UNESCO Quality Physical Education Guidelines for Policymakers awarded the PL concept a central position within their document by highlighting, for instance, that “participation in physical education should support the development of physical literacy and, on the other hand, contribute to global citizenship, through the promotion of life skills and values” (p. 20) [32]. These calls were supported by a recent meta-analysis demonstrating that interventions with an explicit focus on PL can entail positive effects on a range of important outcomes, such as postulated determinants of active lifestyles or the physical activity behaviour per se [33].

Although the most prominent documents of the field set the normative standard to align practices with PL, the holistic claim of the concept brings along certain challenges. For instance, PL is largely missing in documents and guidelines on the national or regional levels [34]. Researchers frequently retain their paradigmatic background and re-interpret the concept to suit their purposes, thus provoking “un-couplings” from the embodiment tenet and the elaborate philosophical underpinnings [35]. Practitioners report difficulties in comprehending the complex conceptualizations, which impedes the necessary applications of real-world settings [36, 37] Similarly, interventions are often not successful in translating PL into appropriate content or show low reporting quality to understand program parameters [18]. Standardized assessments that stand in compatibility with the theoretical foundations of PL are largely lacking, especially for use at scale and with adults [38–40]. Finally, most countries on the globe have not yet gathered information about the alignment of practices with PL [41].

### 1.2 Developmental and global situation of physical literacy

**Fig 1:**
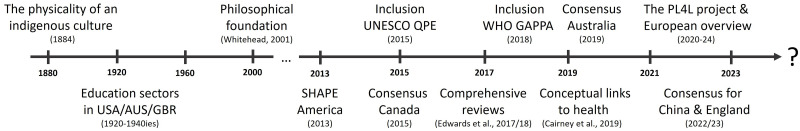
History and milestones of physical literacy research.

The previously listed problems represent a snapshot of current gaps and challenges but are worth embedding into a developmental perspective, as the PL concept has evolved considerably since its first mention in the literature in the 19^th^ century and its academic vitalization approximately 20 years ago [41–44]. Fig 1 illustrates important milestones in the progress of PL (in line with and a continuation of Cairney et al. [42]). However, the literature on PL can so far be characterized as concentrating dominantly on descriptions of the historical evolution (retrospective perspective) or the current situation (cross-sectional perspective). As it is an inherent task of the research system to anticipate future developments and generate innovations to solve problems of the society [45], the PL field can enormously benefit from transcending this retrograde or static focus by a roadmap for its potential development in the future. Indeed, two older articles already embodied a prospective vision [46, 47] but the field has grown considerably during the last eight to ten years [1] and, more importantly, initiatives should detach from the view of single researchers and strive for a more coordinated and overarching effort to define and address the most pivotal actions for PL. Ideally, such a future-oriented perspective capitalizes on a comprehensive representation of PL by bundling different understandings of the concept and converging the majority of cultural as well as paradigmatic perspectives across the world [41, 48]. A cutting-edge vision can also improve its overall impact when replacing a fragmented view with simultaneous consideration of research, practice, and policy aspects [29, 34, 44]. Finally, a PL perspective should also logically involve the broad range of potential settings and application fields mentioned above (i.e., physical education, sport, public health, health care/promotion, rehabilitation, recreation, or community services).

### 1.3 Objectives of the present study

The following questions for research, practice, and policy arise: Which *goals* can be defined for the future development of the field? Which *principles* must be considered, given the specific features of the PL concept? Which concrete *actions* would benefit this development? Which *ways* offer the best opportunity to move the PL field forward?

Therefore, the objective of the present study is to define goals and principles (asking the future-related question *what* is needed?) as well as actions and ways (asking the future-relation question *how* this can be done?) to move the PL field forward worldwide effectively. In the end, this initiative intends to generate a Global Physical Literacy (GloPL) Action Framework that highlights and visualizes the unique challenges and potential solutions of the PL field. In addition, the present endeavour aims to disseminate this action framework in the broad field of physical activity and health [49, 50]. Due to the open format of the questions and goals, we do not specify any empirical hypotheses for this study.

## 2. Methods

### 2.1 Rationale for the study design

Propelled by the advantages of collective intelligence and distributed cognition, this study draws on a group Delphi technique [51] with worldwide representation to generate a GloPL Action Framework. Among the different consensus methods [52–54], the group Delphi represents a “variant in which the anonymity of the experts is abandoned in favour of an open exchange among professionals” [51]. We have chosen this method, as the definition of goals, principles, actions, and ways requires a generative, innovative, inclusive, interactive, and discursive atmosphere. Cutting-edge solutions may come from single experts and minority ideas might gain growing interest when given a platform for argumentation [55]. By contrast, an unvoiced removal of individual ideas at an early stage of the process may undermine this prospectively orientated spirit and simultaneously harm the commitment and ambience within the PL community. Importantly, as the PL field is hallmarked by differing intellectual schools and actor networks [48], we consider it imperative to support a respectful culture and provide the experts with the opportunity to “give contextual justifications for deviating judgments” [54].

**Fig 2:**
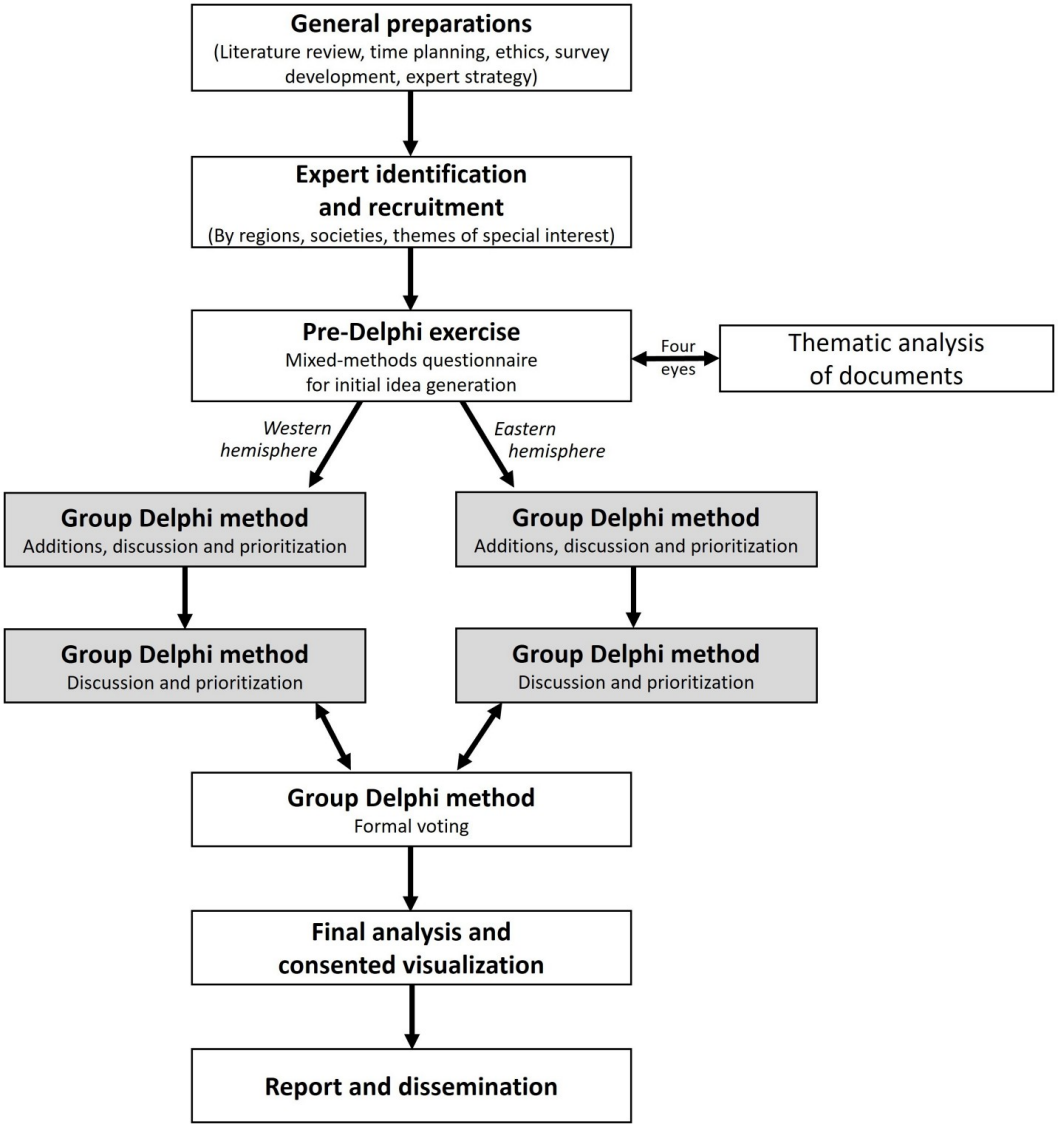
The study design for the development process of the Global Physical Literacy (GloPL) Action Framework. Note: The division of the process into two boxes for the core process of the group Delphi method (i.e., after the pre-Delphi exercise) visualizes the separation in two parallel meetings by hemisphere.

Nevertheless, an open discussion must be combined with structured voting to prioritize actions and extract the most promising solutions. This group Delphi process (see Fig 2) involves the following steps [56, 57]: (a) general preparation (literature review, time planning, ethics, survey development, expert strategy); (b) identification and recruitment of experts; (c) pre-Delphi exercise and subsequent thematic analysis; (d) group Delphi discussion and prioritization; (e) group Delphi voting; (f) final analysis and consented visualization; (g) reporting and dissemination. As the intended global representation makes it unfeasible for practical, financial, and sustainability reasons to meet in person, we will utilize an online format for the group Delphi process. All representatives must provide informed, written consent by filling out a ‘plain language and consent form’ to participate in this study. The entire procedure has undergone ethical review by the Human Ethics Advisory Group Health at Deakin University (sign HEAG-H 06_2024). The reporting of this study protocol follows a detailed, review-supported category system for Delphi techniques in the health sciences [58].

### 2.2 Selection of experts

#### 2.2.1 General ambitions of representation

Three overarching ambitions drive the selection of experts. First, the GloPL Action Framework strives for a *global representation* of PL experts, implying that the selected procedure must adequately consider differing situations worldwide. Second, this study stipulates *themes of special interest*. These representations have the potential to mitigate existing gaps and shortcomings of the specific PL landscape and of physical activity promotion in general, thus facilitating a prospective vision about goals, principles, actions, and ways for the field. Third, this study anticipates the potential diffusion and dissemination of the initiative [49, 50] and, therefore, deliberately includes a representation of *organizations and societies* of the broad physical activity and health area already during the development process. For this study, we define an expert as a person who has considerable *knowledge* and/or *experience* with PL, and/or has the potential to significantly *influence* PL (e.g., via research, regulations, guidelines, or policy) in their country, region, or social networks [59]. We operationalize the knowledge and experience status through thematically relevant research output in both quantity and quality (especially in the previous five years), coupled with visible presence on websites (e.g., in societies, documents, practical initiatives) or in workshops.

On a secondary level, we also take into account the following criteria for the selection of experts: (a) network background or ‘schools of thought’ [51]–the experts should stem from different clusters of researchers (as identified by Young et al. [48]: the ‘idealist embodiment cluster’, the ‘idealist-pragmatic cluster’, the ‘pragmatic health determinant cluster’, and the ‘pragmatic disease prevention cluster’) to not disproportionately favour a specific PL understanding; (b) application field–the experts should occupy the broad range of identified application fields (i.e., physical education, sport, public health, health care/promotion, rehabilitation, recreation, or community services); (c) target group–the work of the experts should cover the entire PL life span (i.e., from early childhood to older adults) and individuals with disabilities or chronic conditions; lastly (d) practice perspective–some representatives should have experience with translations into practice (e.g., physical education, public health initiatives, sport organizations, coaching, interventions). In accordance with the multifaceted nature of PL research and applications, we prioritize expert heterogeneity over homogeneity [60]. All initial invitations will be sent by individualized e-mail (contact information gathered through websites or correspondence addresses in publications). The recruitment of experts will start on March 1^st^, 2024, and end on June 30^th^, 2024, at the latest. In summary, we characterize the applied strategy, in line with the goals and criteria of coverage, as purposeful sampling.

#### 2.2.2 Representatives of geographical regions

**Fig 3:**
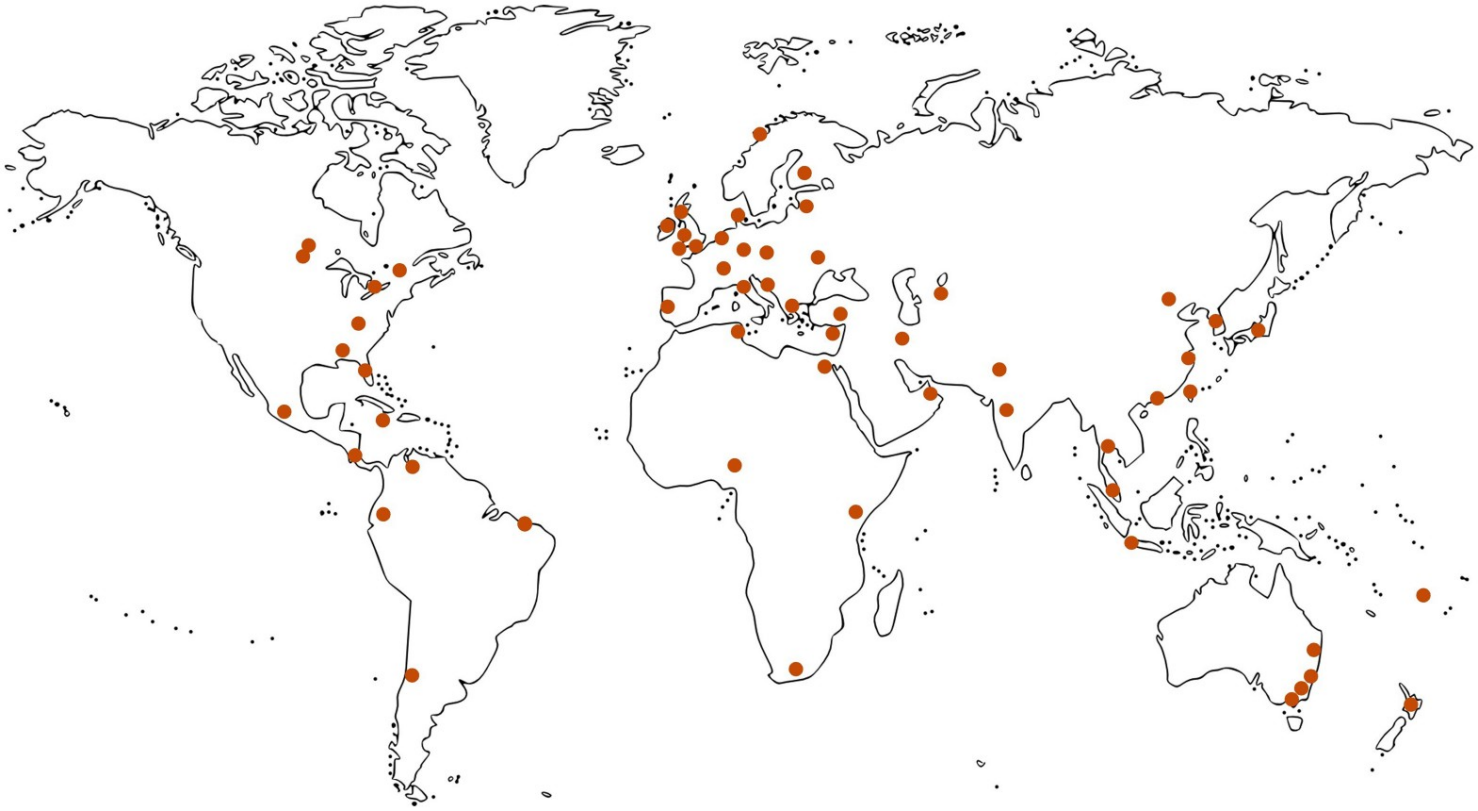
Planned geographical representations for the group Delphi panel (visualization by OpenClipart-Vectors via Pixabay). Note: The specification of the geographical representation bases on the current spread of physical literacy (PL) activities in research, practice, and policy, on the one hand (present situation), but also on the aspiration to cover regions of lower PL development and enable a stronger dissemination of PL physical literacy activities in the future, on the other. At the submission time of this study protocol, geographical representatives have not yet been contacted.

In line with the prospectively oriented vision of the framework, the selection of experts aims to master a trade-off for the specification of geographical representatives. On the one hand, the selection must reflect the geographical focus of current PL activities. Accordingly, the composition of the group Delphi must recognize the progress in North America (Canada, in particular), Europe (Great Britain, in particular), and Australia [2, 6, 34, 41]. On the one other hand, the selection must not neglect entire regions of the world to conceal challenges of conceptual groundwork for certain countries and miss opportunities for developmental work [61]. Therefore, we undertake increasing effort to recruit experts from Africa, South America, and parts of Asia [41, 62]. Given this trade-off, the present authors aim to establish the representations in accordance with an algorithm as depicted in Fig 3 and further explained in S1 Table: North and Middle America (*n* = 10), Australia and Oceania (*n* = 6), Europe (*n* = 19), Africa (*n* = 5), South America (*n* = 4), and Asia (*n* = 15). For the concrete identification of respective representatives, we apply a relative understanding of expertise, implying that the level for final selection must be seen in relation to other actors on the national or regional level (and against the backdrop of the secondary criteria, see section 2.2.1). For the selection of experts, we screen the specific PL publication activities of the respective countries and regions whilst prioritizing activities within the last five years. We have an Eastern European representation with a Ukrainian researcher and consider the war situation with Russia and Belarus. We reject cooperation with individuals from sanctioned regimes as listed by the Department of Foreign Affairs and Trade (DFAT) of the Australian Government.

#### 2.2.3 Representatives of special thematic interest

We will invite seven experts who address topics of special interest or cover typical under-representations of PL initiatives. Specifically, the composition of the group Delphi panel is deliberately extended by advocates for older adults, persons with diseases and disabilities, individuals of low socioeconomic status, as well as indigenous people, as researchers have yielded relatively few perspectives for these target groups [8, 23, 30, 63–66]. We intend to include an expert for urban design to gain inspiration about opportunities to promote PL through the environment [67]. PL in relation to the threat of climate change has only just emerged in the research literature [68] but has not yet influenced the discussion of the field, so we reserve one representation for highlighting the value of integrating ecological aspects for action. For identifying expertise in these niche themes, we also scan PL publication activities in the literature. In accordance with the goal to stress practical perspectives, we finally include one expert who has a strong portfolio in translating PL into real-life applications. In this context, we consult the board of IPLA as a society committed toward practice translation for nominating one expert with such a specific profile. All representatives have full membership status in this process (i.e., including voting) but are additionally invited to make a short thematic statement at the beginning of the discussion process.

#### 2.2.4 Representatives of societies

We proactively offer representatives of organizations and societies, which declare to be inherently committed to promoting physical activity and health, to join this study. The organizations and societies can be assigned to three different categories: from those with an already existing, specific interest in PL; via those with a medium-range interest in promoting physical activity, physical education, or sport science; to those with an overarching focus and broad reach, but for which physical activity represents only one sub-topic. All society representatives actively accompany the study process (including engagement in discussions) and can decide themselves, depending on their specific expertise, whether they feel confident in taking part in the voting. The study team will also integrate an opportunity for this group to skip single questions, with the goal to maximize participation to voting. We apply a tailored request strategy for each organization and society (an initial list can be found in S2 Table). The initial contact will be made via the chairperson, via a board member experienced with PL, or via the lead of a special interest group within the organization. For the process, we recommend the society to nominate one or two persons as representatives who are aware of the mission and vision of the society and simultaneously know PL.

### 2.3 The process

#### 2.3.1 First stage: Pre-Delphi exercise with idea generation

At the beginning, we will send all participants an introductory video (two minutes in length) to explain the entire Delphi group process. Simultaneously, we will provide all participants the plain language and consent form, followed by an initial questionnaire. This questionnaire is split into two parts and slightly varies according to the three representations. The first part involves questions regarding the person (including the represented country/society, the area and duration of expertise, the primary target group of their PL activities, and their application field) as well as the relevance of PL in their country or organization (with two or four closed questions taken from the EUROPLIT study, respectively: dimensions ‘conceptualization’, ‘research’, ‘practical initiatives’, and ‘policy documents’ [34]). The first part serves to describe the experts and composition of the group (and therefore, to re-validate the recruitment strategy) as well as to contextualize the positions organizationally and geographically. In the second part, all participants are asked to express their opinions and ideas on how PL can be significantly moved forward. The introductory statement explicitly invites the experts to cover perspectives from research, practice, and policy. In line with the goals of the study, we specifically inquire about goals and principles (asking *what* is needed?) in a first question and about principles and actions (asking *how* the goals or actions can be addressed?) in a second question. These questions have an open format in which participants can freely report. The representatives are asked to limit each their responses to each question to one page (i.e., 400–500 words).

#### 2.3.2 Second stage: Dissemination and prioritization

The experts will meet face-to-face in the online conference programme Zoom v5.16 (Zoom Video Communications, San José, United States). In line with recommendations regarding the extent of the method [54, 69], we schedule two to three rounds of group Delphi meetings (see grey boxes in Fig 2), each with a duration between 90 and 120 minutes. Due to the distribution of experts across the entire world, we conduct two separate meetings for each round by splitting into a more eastern and a more western hemisphere meeting. However, if experts are unable to realize one appointment, they are welcome to attend the appointment of the other hemisphere. The first author (JCar), who has already orchestrated a consensus process in the PL field [18], will take the moderation role and guide through the discussion.

The first round of meetings will begin with an introductory presentation by the core research team and a repetition of the study goals. We will also establish communication rules (e.g., flat hierarchy and democratic voting principles, appreciating atmosphere, tolerance toward opposing opinions) to ensure adequate social interaction and promote process adherence. Subsequently, the representatives of special interest can provide a short statement for PL development through their thematic lens. The moderator subsequently reports the results of the pre-Delphi exercise by presenting graphics on the identified themes and their assigned relevance (for the analytical procedure, see section 2.4.1). At the same time, the moderator welcomes the representatives to add further (spontaneous) ideas. The initial discussion concentrates on controversial (e.g., those requiring a decision on the direction) and unique themes. At this stage, the experts exchange arguments qualitative reflecting different levels of argumentative depth. Based on the number of identified themes, the moderator pre-defines a maximum time for each topic to not exceed the provided number of Delphi rounds. Depending on the progress, the experts may already participate in an *intermediary voting* after the first round of meetings. For instance, an intermediary voting may serve to gain a better impression of the required attention to be placed on suggestions or find out the favoured rigor of a statement. We may also use the intermediary voting to potentially exclude solutions with low agreement but consider this the latest option to not cut individual contributions at this early stage already (and thus impair motivation and commitment with the study). Such an intermediary voting may take place as a “statistical evaluation in real time” [51] to enable live feedback or between the rounds to harmonize the procedure for the participants of both hemispheric meetings. In any case, we summarize the latest voting at the beginning of each meeting to enable equally informed discussion. If the progress is slower than expected, the moderator may also define smaller working groups with delegated topics or rotating compositions [70]. Between each round, the research team meets (JCar, EM, AM, LB) to analyse the progress and define the goals of the upcoming group Delphi round. All group Delphi meetings are video- and audio-recorded to reconstruct reasons for exclusion, revision, dissent, and inclusion. These recordings represent important process data and can qualitatively complement the subsequent voting data (e.g., by uncovering dynamics in argumentation and interaction).

#### 2.3.3 Third stage: Formal voting

After the second and, if required, after the third round of the group Delphi meeting, we conduct an online voting via Qualtrics (Qualtrics, Provo/Seattle, USA) with all suggested statements that the experts have not sorted out in a previous round (e.g., in an intermediary voting). The goal of the formal voting is to consensually establish the consideration or non-consideration of themes within the final GloPL Action Framework. In the second round, at least two thirds (i.e., ≥ 66.7%) of all representatives must positively agree with one theme to be finally included in the consensus [71]. All statements below a positive agreement value of 25% are excluded. We retain themes with an agreement rate between 25% and 66.7% for adjustment or further discussion. These themes are taken into a third group Delphi round, where the final voting is based on simple majority (i.e., inclusion of themes with an agreement ≥ 50% and exclusion of themes with an agreement <50%).

**Fig 4:**
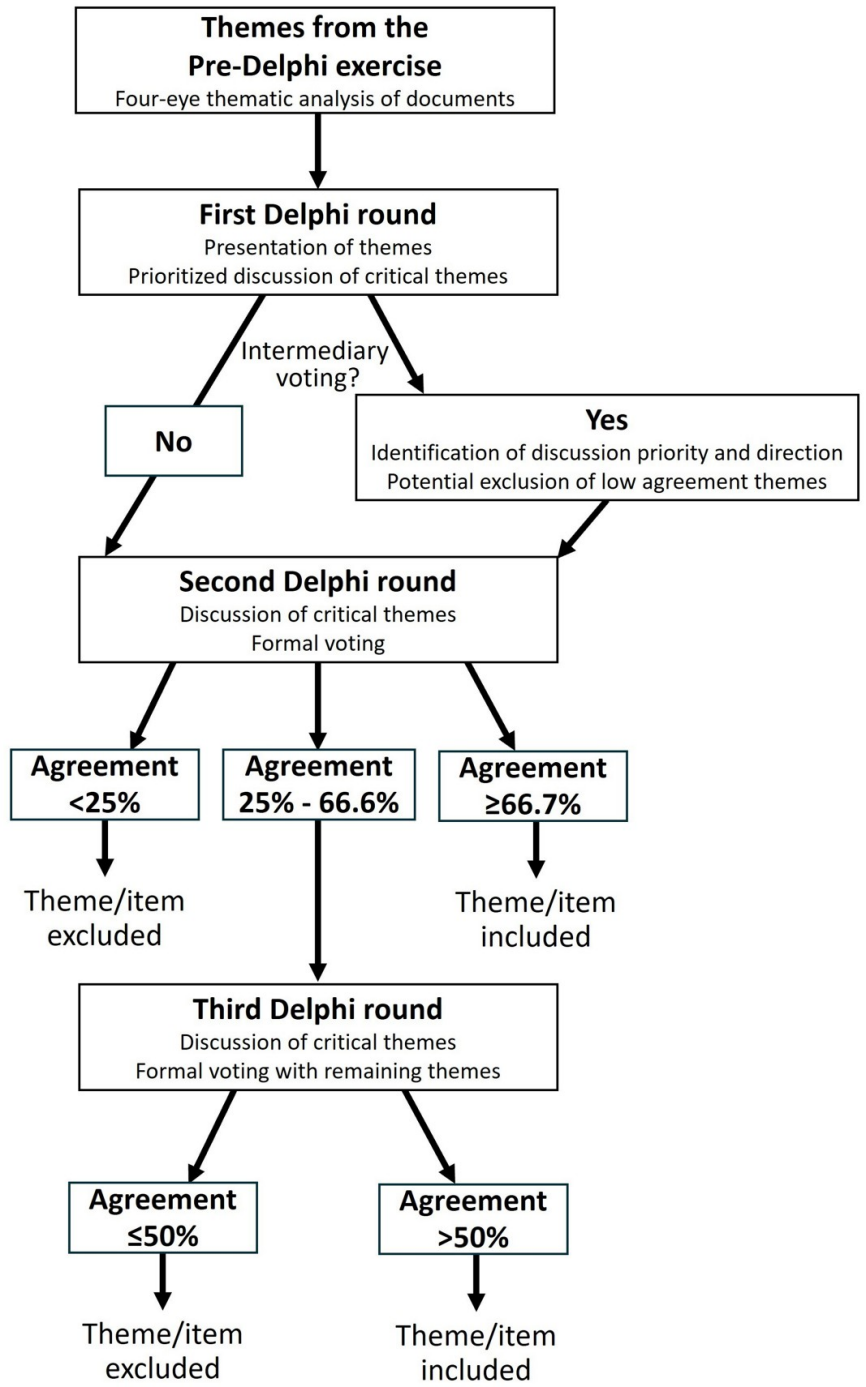
Process and decision tree for the voting.

Two formats come into question for the formal voting. First, questions can take a *nominal format* with the response options ‘I agree with this statement’ versus ‘I do not agree with this statement’. For single questions, we may also introduce a response option with a plead for revision (e.g., ‘I prefer to revise this statement’ or ‘I agree with this statement if revised’). Second, questions can take an *ordinal format* when the focus is not placed on the basic inclusion versus non-inclusion but on the favoured rigor of a statement or recommendation (e.g., as response options: ‘has to include’, ‘should include’, ‘should be checked for compatibility’, or ‘do not suggest any prescription’). In such a case, the expert panel prefers the option that, in a descending order, still meets approval of at least two thirds of the members [71]. For psychometric reasons, we attempt to limit the number of selectable responses to three or at maximum four [72]. A comment box at the end of the online survey allows experts to freely comment on response options or give reasons for their voting. The project members AM and MN will pilot test the developed survey instruments and provide feedback (e.g., on face validity and comprehensibility). Fig 4 summarizes the stage-dependent decision mechanisms in a flow chart.

Combined, ‘*consensus*’ in this study means that the framework nominates goals, principles, actions, and ways for PL development that two thirds of the experts at an earlier stage of the discussion process consider worth following or, after more controversial and extensive discussion, at least the half of all experts (simple majority rule) consider worth following. In absence of basically successful consensus after the third meeting and second round of formal voting, respectively, the core research team (JCar, EM, AM, LB) will reflect on how to proceed (i.e., potential stopping criterion) and communicates the next steps to the entire panel of representatives. All responses, either in discussions or final reports, are presented anonymously. However, we explicitly ask participants’ names in the survey for organization reasons, as we send a personalized reminder mail after the deadline to give the opportunity to fill for two additional weeks. Afterwards, we irreversibly close the online survey. We exclude experts from final report if they miss more than one formal voting. An entire formal voting round is considered invalid, if less than 66.7% of the remaining geographical and special interest representatives fill the survey.

### 2.4 Data analysis and presentation

#### 2.4.1 Pre-Delphi exercise with idea generation: Thematic analysis

Technically, the open-format reports from the pre-Delphi exercise constitute written text. We submit these texts to document analysis, which characterizes “a systematic procedure for reviewing or evaluating documents–both printed and electronic (computer-based and Internet-transmitted) material” [73]. Document analysis involves the following phases: (a) reading the texts; (b) extracting themes; (c) analysing data; and (d) distilling findings [74]. Nevertheless, document analysis is not a rigid and axiomatically linear process, which means that loops in the process may benefit pattern recognition [75]. Two researchers from the team familiarize with the data by first skimming and then thoroughly reading each report (four-eye principle) [73, 76]. Both researchers independently identify interesting features and code passages within each report [76]. As typical for thematic analysis, these features and codes can reflect a different degree of argumentative depth. Afterwards, the two researchers inductively derive themes across the documents through structured comparison [77]. This process as the core of thematic document analysis “leads to moving from specific observations to social constructions, theories, and broader generalizations” (p. 392) [75] relevant to generate a GloPL Action Framework. After independent work, the two researchers will meet to aggregate the two separate analyses, including the provision of adequate names for the themes [76]. Disagreements are solved by verbal consensus. Extending the thematic analysis by a quantitative content analysis, we will translate the aggregated themes into descriptive statistics across all reports (e.g., absolute and relative frequency) and visualize the data through diagrams to facilitate the entry into the discussions and not initiate an idea generation process in the group format from scratch. As document analyses are often harmonized with other data collection methods [73], we qualify the combination of an initial document analysis with subsequent voting (as part of the group Delphi), from a data analytical standpoint, as a sequential mixed-methods endeavour [78].

#### 2.4.2 Voting

We will calculate descriptive statistics for the voting patterns of each group Delphi round. All nominal and ordinal responses are presented in both absolute and relative numbers. For ordinal responses, we additionally compute accumulated agreement values (in descending order). The statistics ground only on validly submitted reports and do not include experts without submission within the extended deadline. All statements to be included in the final framework (1 = inclusion, 0 = exclusion) must strictly surpass the pre-defined thresholds according to the respective stage of the group Delphi process (see section 2.3.3 and Fig 4). The included statements enter the subsequent visualization stage. Intermediary voting processes (i.e., for providing direction for opposing suggestions or a deliberate reduction in the number of themes) and further analyses (e.g., response behaviour according to continents) have an informal or orientating function and are, in concert with the flexible nature of the Delphi method, designed by purpose. However, these additions can only lead to the exclusion of a theme (e.g., in case of a too high number of themes for adequate handling) but not to a final inclusion, as this step requires formal voting.

#### 2.4.3 Visualization

In addition to descriptive statistics, all findings will be illustrated in a figure to facilitate the communication and dissemination of the most crucial tenets of the Global Physical Literacy Action Framework (see here for an output inspiration in the physical education [79]). The differentiation into goals and principles, on the one hand, as well as actions and ways to move PL globally forward, on the other, may inform the construction of the target illustration. To master the trade-off between parsimony/clarity and thematic completeness, it may be useful to bundle certain consensually defined outcome categories. Although the core research team (JCar, EM, AM, LB) will develop suggestions for visualization, all representatives are given the opportunity to inspect the figures and file objections. In case of multiple figure versions, the representatives are asked to indicate the favoured variant (decision by simple majority).

### 2.5 Moderation and analysis of opportunities and challenges

Although the Delphi technique is nowadays well accepted [71], the literature still discusses advantages and disadvantages of the method [51, 52]. To ensure an efficient and straightforward process while maximizing the probability of achieving a successful consensus on the most important future directions for the PL field, we undertook an explicit reflection task about typical challenges and opportunities specific to the group Delphi process (see S3 Table).

### 2.6 Reporting and dissemination

The entire group (total number of experts resulting from the representations: 70–84) will release a final statement of the GloPL Action Framework. This final statement encompasses the visual representation as well as a textual elaboration of the included themes and recommendations. All representatives of the organizations and societies are invited to transport this statement to their society’s official committee in the phase of developing the draft (for a preliminary list of societies, see again S2 Table). If endorsed, the society can co-release the statement with the representatives. We aim to present the findings at conferences on both international and national levels. In accordance with an inclusive gesture and a decentralized dissemination approach [80], we will prepare general presentation material that the representatives can use for their communication purposes after their specific adjustment (to guarantee compatibility with the intended context). Finally, we outline the development process and outcomes of the GloPL Action Framework in peer-reviewed publications. From a thematic standpoint, the scope of the journal should harmonize with the broad application contexts of PL (e.g., physical education, sport, public health, health care/promotion, rehabilitation, recreation, or community services). We give all representatives the opportunity to contribute to the central publication of the entire group and to be listed as a co-author (if their contribution merits that of authorship as per the guidelines of the target journal). The final reporting of the development process and outcomes adheres to the Recommendations for the Conducting and REporting of DElphi Studies (CREDES). In case of the successful development of a GloPL Action Framework, we will align the diffusion and dissemination process, including its suggested strategies and determinants, with an established theoretical framework [49].

## 3. Discussion

Despite registerable progress in research activities, practices, and policies on PL in recent years, initiatives on the concept largely characterize a fragmented organization without adherence to an explicit, shared roadmap toward future development. This manuscript a-priori explains the process that aims to close this gap by following the goal to develop a GloPL Action Framework. This article describes the upcoming process with relevant background information, decisions mechanisms, and reasons in an extent that could have hardly been realized in an original results article. From a scientific integrity and transparency standpoint, it is important that such comprehensive expert processes follow pre-defined decision criteria to counteract suspicions of deliberate influence. In this regard, external readers and followers of this study on the global level have the potential to better interpret future decisions and elements of the framework. Methodically embedded into a group Delphi technique, this study ensures broad coverage of experts through an algorithm of global representation, the definition of seven special interest themes, and an inclusion of representations of organizations and societies. The outcomes of this study have, for instance, the potential to emphasize major challenges for PL research, vocalize suggestions for treating differing PL conceptualizations, define priority for assessment and intervention issues, improve the transfer into practice, or strengthen connections to policy. Most importantly, the process of developing this framework is open in terms of cutting-edge solutions that may have not been voiced so far.

We mention the following limitations for this study within this protocol. First, it is for time shift reasons not feasible to schedule a meeting with all representatives simultaneously. The separation into two time zones may result in discussions (e.g., on the treatment of non-identical definitions) taking differing directions. As cutting-edge solutions may have to be argumentatively empowered by single experts, such a separation may also cause non-advocacy for such a solution in the parallel meeting without the respective expert. Second, the accentuation of solutions depends on the rhetoric skills, self-confidence, and aura of the experts; shyer representatives and experts with lower English-speaking capabilities might have difficulties effectively expressing their standpoint. In this regard, the moderator is going to encourage equal voice for every participant and introduces the group Delphi technique with a call for tolerance and appreciation among representatives. The actual voting might be less affected due to the opportunity to use translation tools during the survey. Third, we cannot exclude that the research team with its academic background and cultural experiences might affect the organization of the study and the thematic analysis. Fourth, the group Delphi technique may be considered resource-intensive (albeit announced as being less intensive than a traditional Delphi [51]) and lacking commitment. Against this background, we write personalized e-mails, transparently describe the involvement in this study protocol, and cultivate an inclusive atmosphere with the opportunity to co-produce the final statement, publication, and dissemination.

Nevertheless, the GloPL Action Framework can transcend the existing cross-sectional or historical perspectives on PL by a prospective roadmap for future goals and actions across the world. Specifically, this study takes an integrating approach to PL development by converging research, policy, and practice perspectives. PL—often portrayed as a holistic framework for lifelong adoption of a physically active lifestyle—has the potential to significantly extend practices of physical activity in a range of application fields (e.g., enriching experiences in physical education, meaningful sport involvement, better contributions to public health, person-centred health care/promotion, biopsychosocial rehabilitation, more pleasant recreation, or ‘activating’ community services). In this regard, the present study assumes that generating a specific framework to energize PL over the globe merits strong value for physical activity promotion and may mark a milestone for the concept.

## Supporting information

S1 Table Except of organizations relevant for the broad area of physical activity and health. (DOCX)

S2 Table Organizations and societies in the wide area of physical activity and health with potential interest in promoting physical literacy. (DOCX)

S3 Table Overview of challenges, the respective reflection and adopted measures, and potential opportunities of the group Delphi process toward the development of a Global Physical Literacy Action Framework. (DOCX)

## Abbreviations

GloPL Action Framework (project acronym): Global Physical Literacy Action Framework
PL: Physical Literacy
UNESCO: United Nations Educational, Scientific and Cultural Organization
WHO: World Health Organization
