# Supplementary material for: Development of a Global Physical Literacy (GloPL) Action Framework: Study protocol for a consensus process
Source: PLoS One. 2024 Aug 12;19(8):e0307000. doi: 10.1371/journal.pone.0307000 (PMC11318864; doi:10.1371/journal.pone.0307000)
Supplement: S1 Table — (DOCX) [file pone.0307000.s001.docx]

**S1 Table.** Except of organizations relevant for the broad area of physical activity and health.

| **Continent** | **Country/region** | **Planned number of representatives** | **Comment and/or included countries per region** |
| --- | --- | --- | --- |
| EUROPE | United Kingdom / Great Britain | 4 | “Motherland” of physical literacy, many activities, consensus statements, state-wide organization (England, Wales, Scotland, Northern Ireland) |
|  | Ireland | 1 | Progress in PL activities |
|  | German-speaking countries | 1 | Germany, Switzerland, Austria, Liechtenstein |
|  | France | 1 | Including Monaco |
|  | Italy | 1 | Including San Marino and Holy See |
|  | Iberian countries | 1 | Spain, Portugal, Andorra |
|  | Denmark | 1 | Non-anglophone country with considerable progress in physical literacy, own consensus |
|  | Northern countries | 2 | Sweden, Finland, Norway, Iceland, Greenland, Faroe Islands |
|  | Be-Ne-Lux countries | 1 | Belgium, Netherlands, Luxembourg |
|  | Central Europe | 1 | Czechia, Hungary, Poland, Slovakia, Slovenia |
|  | Baltic countries | 1 | Lithuania, Latvia, Estonia |
|  | Balkan countries | 2-3 | Albania, Bosnia and Herzegovina, Bulgaria, Greece, Kosovo, Montenegro, North Macedonia, Croatia, Serbia |
|  | Eastern Europe* | 1 | Ukraine, Moldova, Romania |
|  | Mediterranean countries | 0-1 | Cyprus, Malta |
| NORTH AND MIDDLE AMERICA | Canada | 4 | Leading academic country, many practical initiatives related to physical literacy |
|  | United States of America | 3 | Large country with several academic and practical initiatives related to physical literacy |
|  | Mexico | 1 | Large country with development potential for physical literacy |
|  | Caribbean countries | 1 | Jamaica, Cuba, Trinidad and Tobago, Antigua and Barbuda, Bahamas, Barbados, Dominica, Dominican Republic, Grenada, Guyana, Haiti, Saint Kitts and Nevis, Saint Lucia, Saint Vincent and the Grenadines |
|  | Middle America mainland | 1 | Costa Rica, Panama, Nicaragua, Guatemala, Honduras, El Salvador, Belize |
| SOUTH AMERICA | Brazil | 1 | Large country with development potential for physical literacy |
|  | North | 1 | Colombia, Venezuela, Guyana, French Guiana, Suriname |
|  | West | 1 | Peru, Bolivia, Ecuador |
|  | South | 1 | Argentina, Chile, Uruguay, Paraguay |
| ASIA | China | 2 | Large country with many physical literacy initiatives, recent consensus process |
|  | India | 1-2 | Large country with a growing number of physical literacy initiatives |
|  | Japan | 1 | Large, industrialized country with development potential for physical literacy |
|  | East Asia | 1-2 | South Korea, Taiwan, North Korea, Mongolia |
|  | West Asia | 2-3 | Türkiye, Bahrain, Kuwait, Oman, United Arab Emirates, Yemen, Armenia, Azerbaijan, Georgia, Iran, Iraq, Palestine, Syria, Saudi Arabia, Qatar, Israel, Jordan, Lebanon |
|  | Central Asia | 1 | Kazakhstan, Kyrgyzstan, Tajikistan, Turkmenistan, Uzbekistan |
|  | Hong Kong | 1 | Very strong academic progress |
|  | South-Central Asia | 1 | Pakistan, Afghanistan, Bhutan, Maldives, Sri Lanka, Bangladesh, Nepal |
|  | South-Eastern Asia | 2-3 | Brunei, Cambodia, East Timor, Indonesia, Laos, Malaysia, Myanmar, Philippines, Singapore, Thailand, Vietnam |
| AFRICA | Northern East | 1 | Eritrea, Sudan, Chad, Egypt, Libya |
|  | Northern West | 1 | Algeria, Morocco, Tunisia, West Sahara, Mauritania, Mali, Niger, Senegal, Gambia |
|  | Central West | 1 | Benin, Burkina Faso, Cape Verde, Ivory Coast, Ghana, Guinea, Guinea-Bissau, Liberia, Nigeria, Sierra Leone, Togo, Angola, Cameroon, Central African Republic, DR Congo, Equatorial Guinea, Gabon, Congo-Brazzaville, Sao Tome and Principe |
|  | Central East | 1 | Burundi, Comoros, Djibouti, Ethiopia, Kenya, Madagascar, Malawi, Mauritius, Mozambique, Rwanda, Seychelles, Somalia, South Sudan, Uganda, Zambia, Tanzania |
|  | Southern | 1 | South Africa, Lesotho, Botswana, Eswatini, Namibia, Zimbabwe |
| AUSTRALIA AND OCEANIA | Australia | 4 | Many academic and practical initiatives related to physical literacy |
|  | New Zealand | 1 | Large country with an own culture and understanding of physical literacy |
|  | Pacific islands | 1 | Fiji, Papua New Guinea, Solomon Islands, Vanuatu, Micronesia, Kiribati, Marshall Islands, Nauru, Palau, Cook Island, Niue, Samoa, Tonga, Tuvalu |

Note: The selection bases on the current status of PL activities, on the one hand, but to enable global representation for future development, on the other; some countries are lifted out from regions (e.g., Denmark from Scandinavia or China and Japan from East Asia), as they are awarded an own status (rationale is given).
*We exclude Russia and Belarus for political reasons, as we plan to have an East European representation with a Ukrainian researcher and most western countries (e.g., United States, Great Britain, Germany) avoid collaborations after the start of the war in February 2022.
