# Supplementary material for: Development of a Global Physical Literacy (GloPL) Action Framework: Study protocol for a consensus process
Source: PLoS One. 2024 Aug 12;19(8):e0307000. doi: 10.1371/journal.pone.0307000 (PMC11318864; doi:10.1371/journal.pone.0307000)
Supplement: S2 Table — (DOCX) [file pone.0307000.s002.docx]

**SF2 Table.** Organizations and societies in the wide area of physical activity and health with potential interest in promoting physical literacy.

| **Nr.** | **Abbreviation** | **Full name** | **Rationale** |
| --- | --- | --- | --- |
| 1 | WHO | World Health Organization | Umbrella organization for global health, physical activity as an important health topic, global action plan on physical activity 2018-2030 (has included physical literacy) |
| 2 | UNESCO | United Nations Educational, Scientific and Cultural Organization | Umbrella organization for global education, physical education as an important subject, quality physical education guidelines for policymakers (has included physical literacy) |
| 3 | HEPA Europe | European network for the promotion of health-enhancing physical activity | Important network for physical activity and health, under the umbrella of the World Health Organization (“provide a forum for the advancement of health-enhancing physical activity research, policy and practice for better health and well-being” across Europe) |
| 4 | AIESEP | Association Internationale des Écoles Supérieures d'Éducation Physique | Important association at the intersection of physical education and health (“We promote effective physical and health education, physical activity and sport participation across the lifespan.”) |
| 5 | ISCA | International Sport and Culture Association | Important association for recreational sport and physical activity (“We empower organisations worldwide to enable citizens to enjoy their human right to move”) |
| 6 | EUPEA | European Physical Education Association | Important association for physical education (“Promote and defend physical education as an important educational area within the school system and the training of young people”) |
| 7 | ICSSPE | International Council of Sport Science and Physical Education | Important association in the broad field of sport science (“maintaining an inclusive perspective on the different disciplines of sport, sport science and physical education”) |
| 8 | IAHPEDS | International Alliance for Health, Physical Education, Dance, and Sport | Important network in the broad field of health and physical education (“Advance international research, learning, practices and standards […] to motivate individuals to adopt healthy, physically active lifestyles”) |
| 9 | FIEPS | Fédération Internationale d'Education Physique et Sportive | Important federation to promote physical activity in many spheres (“to promote, in all countries, the development of physical, educational, recreational and leisure activities, for the training of teachers”) |
| 10 | ASPA | Asia-Pacific Society for Physical Activity | Important society for physical activity (“to advance the science and practice of physical activity in Australasia through education and research”) |
| 11 | ACSM | The American College of Sports Medicine | Important society for sport medicine and exercise science (“advances and integrates scientific research to provide educational and practical applications of exercise science and sports medicine”) |
| 12 | ISPAH | International Society for Physical Activity and Health | Important society for physical activity and health “To advance and promote physical activity as a global health priority through excellence in science (research), education, capacity building and advocacy” |
| 13 | ISBNPA | International Society of Behavioral Nutrition and Physical Activity | Important society for changing physical activity behavior (“We stimulate and promote innovative and impactful research in behavioral nutrition and physical activity to improve human and planetary health and well-being worldwide”) |
| 14 | NASPSPA | North American Society for the Psychology of Sport and Physical Activity | Important society in the field of human physical activity behavior (“develop and advance the scientific study of human behavior when individuals are engaged in sport and physical activity; facilitate the dissemination of information […]”) |
| 15 | NASPEM | North American Society for Pediatric Exercise Medicine | Important society for the promotion of exercise in children and adolescents (“to promote exercise science, physical activity, and fitness in the health and medical care of children and adolescents”) |
| 16 | IMDRC | International Motor Development Research Consortium | Important network for motor development research (“To increase the visibility and impact of Motor Development research over the next decade through meaningful collaborations [… and to ensure that] all people reach their fullest potential to live an active and healthy life through movement”) |
| 17 | AFPAN | African Physical Activity Network | Important network for physical activity on the African continent (“to evaluate models of best practice which are regionally appropriate and meaningful through evidence-based research in order to promote health through sport and related activities in the African region”) |
| 18 | RAFA-PANA | Physical Activity Network of the Americas | Important network on physical activity in South America (“to integrate, strengthen and disseminate the policies, strategies and experiences of local and national networks for the promotion of a healthy lifestyle by encouraging the regular practice of physical activity”) |
| 19 | ACPES | ASEAN Council of Physical Education and Sport | Important society for physical education and sport in the ASEAN region (“to promote communication, interaction, and cooperation among ASEAN physical and sport education professionals and students in educational, scientific, and research institutions”) |
| 20 | SHAPE America | Society of Health and Physical Educators | Important society for health and physical education professionals (“To advance professional practice and promote research related to health and physical education, physical activity, dance and sport”) |
| 21 | IPLA | International Physical Literacy Association | Specific network to promote physical literacy across the world (“Facilitate and support the adoption and promotion of physical literacy in communities worldwide”) |
| 22 | IPLC | The International Physical Literacy Conferences | Specific network to promote the exchange on physical literacy (“to bring together health, education, recreation and sport experts to advance the knowledge, application and implementation of physical literacy programming across the globe”) |

Note: The broad area of physical activity and health encompasses (among others) the following application fields: physical education, sport, health care, rehabilitation, recreation, and community services.

**Table 3.** Overview of challenges, the respective reflection and adopted measures, and potential opportunities of the group Delphi process (51) toward the development of a Global Physical Literacy Action Framework.

| **Discussed challenges** | **Reflection on the challenges and respective measures taken (by the research team) to face these challenges** | **Opportunities** |
| --- | --- | --- |
| Coverage of the whole range of opinions by the limited number of experts | Deliberate selection of experts covering different geographical regions (see Figure 3 and Supplementary Table 1) and schools of thought (see sections 2.2.1 and 2.2.2); the importance of a heterogeneous coverage is prioritized over a limited number of experts | Recording of majority and minority votes |
| Selection and focus on a few, central topics/questions | Formulation of clear goals and research questions (see section 1.3); the distinction into goals/principles (*what* has to be addressed) and actions/ways (*how*?) helps focus the study; an intermediary voting can help concentrate the number of themes (e.g., by sorting out very extravagant solutions) at a comparably early stage; structured voting (sections 2.3.3 and 2.4.2, figure 4) | Identification of areas of consensus or dissent in the case of divergent expert judgments |
| Willingness of relevant experts to participate | Individualized contact by mail (section 2.2.1); advertisement for a straightforward process with an ambitious team; deliberate absence of a formalized steering/advisory committee to signalize space for co-creation (open process); considerable opportunity for networking; broad dissemination strategy (sections 2.2.1, 2.2.4, and 2.6); opportunities to participate in terms of publication and dissemination (section 2.6) | Direct exchange between experts from different disciplines and institutional affiliations (inter-/transdisciplinary) |
| No “representativeness” of the results | Broad recruitment strategy (sections 2.1, 2.2 and figure 3); definition of a structured process (section 2.3) with a particular focus on addressing the most critical discussion points | Reduction of uncertainty within the expert group |
| Opinion leaders and window-dressing (special moderation skills required) | Accumulation of experience with moderating consensus projects (e.g., PLIRT study); specific advice in terms of the methods and moderation (MN, AS); establishment of communication rules with flat hierarchy (section 2.3.2); a-priori definition of a maximum time for each topic, which is linked to the fact that not each representative can speak excessively (section 2.3.2); reasons for exclusion, revision, dissent, and inclusion (section 2.4.2.) | Clarification of reasons for dissent (e.g., factual of semantic reasons) |
| Risk of instrumentalization of the procedure, especially in the case of politically and socially contested issues | From a political perspective, the topic is relatively neutral; risk of instrumentalization is given if researchers hope to better profile for their approaches or topics and in terms for some special themes (sections 2.2.1 and 2.2.3); mitigation through individualized mail contact (section 2.2.1) and a clarification of roles (section 2.3.2) | Relatively fast procedure (especially compared to the classical Delphi procedure) |
| Limited scope of forecasts due to uncertain possible futures | The aim of the project is exactly to provide a roadmap for future physical literacy activities and, therefore, reduce uncertainty for the field (sections 1.2 and 1.3) | High connectivity in the research process |

Note: The challenges and opportunities are retrieved from a text group discussing the group Delphi method (51); acronyms are retrieved from the authors list of this article.
Abbreviation: PLIRT = Physical Literacy Interventions Reporting Template.
