# Supplementary material for: Development of a Global Physical Literacy (GloPL) Action Framework: Study protocol for a consensus process
Source: PLoS One. 2024 Aug 12;19(8):e0307000. doi: 10.1371/journal.pone.0307000 (PMC11318864; doi:10.1371/journal.pone.0307000)
Supplement: S3 Table — (DOCX) [file pone.0307000.s003.docx]

**SF3 Table.** Overview of challenges, the respective reflection and adopted measures, and potential opportunities of the group Delphi process (51) toward the development of a Global Physical Literacy Action Framework.

| **Discussed challenges** | **Reflection on the challenges and respective measures taken (by the research team) to face these challenges** | **Opportunities** |
| --- | --- | --- |
| Coverage of the whole range of opinions by the limited number of experts | Deliberate selection of experts covering different geographical regions (see Figure 3 and SF1 Table) and schools of thought (see sections 2.2.1 and 2.2.2); the importance of a heterogeneous coverage is prioritized over a limited number of experts | Recording of majority and minority votes |
| Selection and focus on a few, central topics/questions | Formulation of clear goals and research questions (see section 1.3); the distinction into goals/principles (*what* has to be addressed) and actions/ways (*how*?) helps focus the study; an intermediary voting can help concentrate the number of themes (e.g., by sorting out very extravagant solutions) at a comparably early stage; structured voting (sections 2.3.3 and 2.4.2, figure 4) | Identification of areas of consensus or dissent in the case of divergent expert judgments |
| Willingness of relevant experts to participate | Individualized contact by mail (section 2.2.1); advertisement for a straightforward process with an ambitious team; deliberate absence of a formalized steering/advisory committee to signalize space for co-creation (open process); considerable opportunity for networking; broad dissemination strategy (sections 2.2.1, 2.2.4, and 2.6); opportunities to participate in terms of publication and dissemination (section 2.6) | Direct exchange between experts from different disciplines and institutional affiliations (inter-/transdisciplinary) |
| No “representativeness” of the results | Broad recruitment strategy (sections 2.1, 2.2 and figure 3); definition of a structured process (section 2.3) with a particular focus on addressing the most critical discussion points | Reduction of uncertainty within the expert group |
| Opinion leaders and window-dressing (special moderation skills required) | Accumulation of experience with moderating consensus projects (e.g., PLIRT study); specific advice in terms of the methods and moderation (MN, AS); establishment of communication rules with flat hierarchy (section 2.3.2); a-priori definition of a maximum time for each topic, which is linked to the fact that not each representative can speak excessively (section 2.3.2); reasons for exclusion, revision, dissent, and inclusion (section 2.4.2.) | Clarification of reasons for dissent (e.g., factual of semantic reasons) |
| Risk of instrumentalization of the procedure, especially in the case of politically and socially contested issues | From a political perspective, the topic is relatively neutral; risk of instrumentalization is given if researchers hope to better profile for their approaches or topics and in terms for some special themes (sections 2.2.1 and 2.2.3); mitigation through individualized mail contact (section 2.2.1) and a clarification of roles (section 2.3.2) | Relatively fast procedure (especially compared to the classical Delphi procedure) |
| Limited scope of forecasts due to uncertain possible futures | The aim of the project is exactly to provide a roadmap for future physical literacy activities and, therefore, reduce uncertainty for the field (sections 1.2 and 1.3) | High connectivity in the research process |

Note: The challenges and opportunities are retrieved from a text group discussing the group Delphi method (51); acronyms are retrieved from the authors list of this article.
Abbreviation: PLIRT = Physical Literacy Interventions Reporting Template.
